# Root hair growth from the pH point of view

**DOI:** 10.3389/fpls.2022.949672

**Published:** 2022-07-27

**Authors:** Anett Stéger, Michael Palmgren

**Affiliations:** Department of Plant and Environmental Sciences, University of Copenhagen, Copenhagen, Denmark

**Keywords:** root hair, tip growth, polar growth, pH, ROS, Ca^2+^, plasma membrane H^+^ -ATPase, protons

## Abstract

Root hairs are tubular outgrowths of epidermal cells that increase the root surface area and thereby make the root more efficient at absorbing water and nutrients. Their expansion is limited to the root hair apex, where growth is reported to take place in a pulsating manner. These growth pulses coincide with oscillations of the apoplastic and cytosolic pH in a similar way as has been reported for pollen tubes. Likewise, the concentrations of apoplastic reactive oxygen species (ROS) and cytoplasmic Ca^2+^ oscillate with the same periodicity as growth. Whereas ROS appear to control cell wall extensibility and opening of Ca^2+^ channels, the role of protons as a growth signal in root hairs is less clear and may differ from that in pollen tubes where plasma membrane H^+^-ATPases have been shown to sustain growth. In this review, we outline our current understanding of how pH contributes to root hair development.

## Introduction

Root hairs are single tubular extensions of root cells that enhance water and nutrient uptake by increasing the root surface area. Furthermore, they facilitate beneficial microbial interactions. Root hairs develop from a subset of epidermal cells, the trichoblasts, but not from the hairless atrichoblasts. The fate of root epidermal cells seems to be determined at an early stage of development as trichoblasts are distinguishable from atrichoblasts prior to the emergence of the root hair, partially due to their shorter cell length (Dolan et al., 1994; Masucci et al., 1996). When epidermal cells enter the maturation zone, root hair-promoting transcription factors are activated and then the root hair initiation site is determined (Balcerowicz et al., 2015).

**Figure 1:**
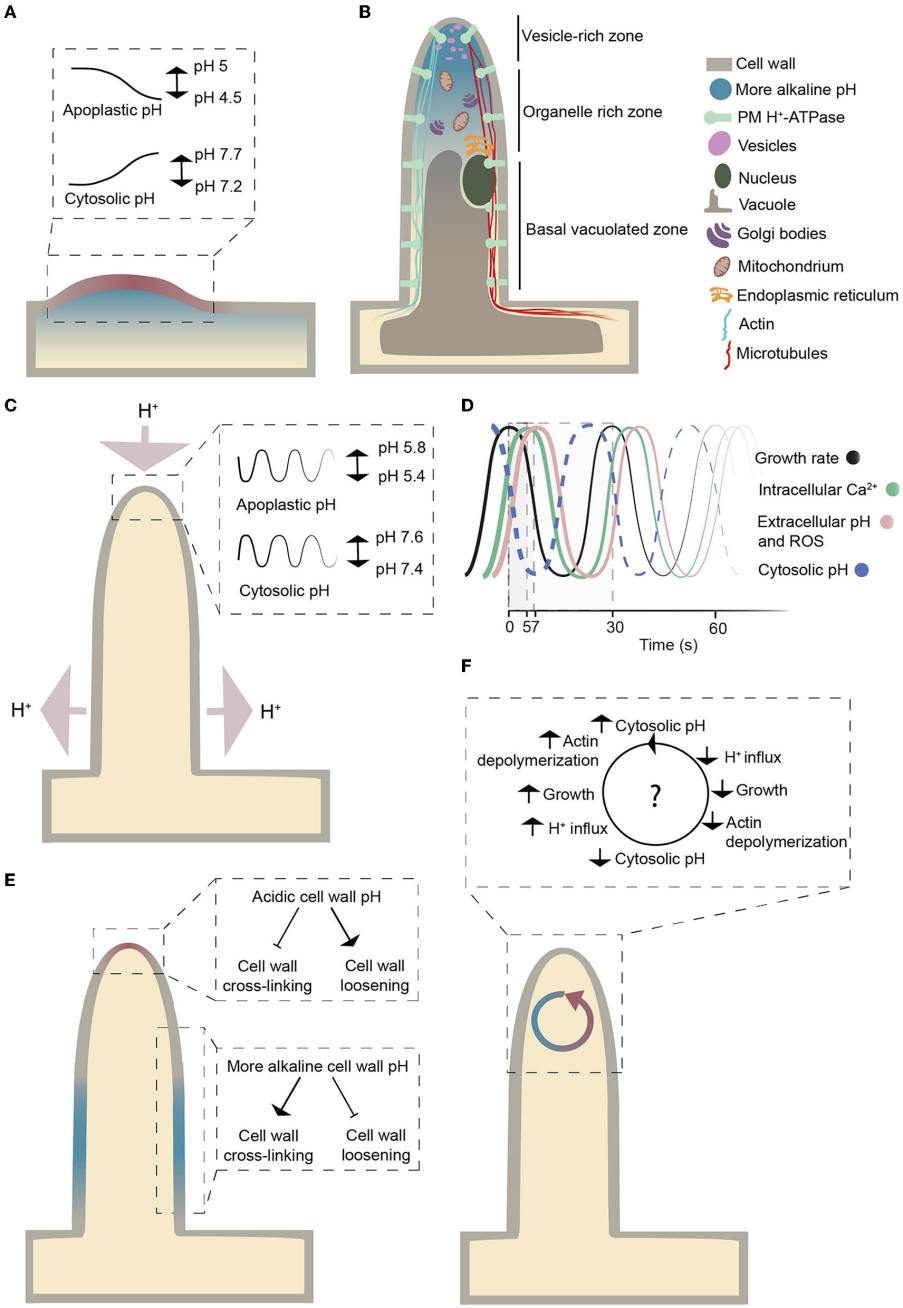
Schematic representation of a growing root hair. **(A)** The pH changes during bulge formation in Arabidopsis. The pH values are based on data in Bibikova et al. (1998). Colors indicate a localized pH increase (blue) and decrease (red) at the site of initiation in trichoblasts. **(B)** Schematic representation of the cytosolic pH gradient and the organelles within a growing root hair. The figure is adapted from Datta et al. (2011). A more alkaline cytosolic pH at the root hair tip compared to the base reflects measurements by Bai et al. (2014). **(C)** Graphical illustration of proton fluxes and oscillatory pH changes at the tip during root hair growth. Light purple arrows indicate net proton fluxes. The pH values are based on the analysis of Monshausen et al. (2007). **(D)** Growth rate, intracellular Ca^2+^, extracellular pH, and ROS oscillations on a time scale. The figure is adapted from Cárdenas (2009). Time values are from Monshausen et al. (2007) and Monshausen et al. (2008). The dashed curve represents the expected cytosolic pH oscillations. **(E)** Graphical illustration of how the cell wall pH could influence cell wall cross-linking. The figure is adapted from Schoenaers et al. (2017). **(F)** Overview of how cytoplasmic pH changes might influence actin polymerization and depolymerization based on the hypothesis from Lovy-Wheeler et al. (2006).

Several events during root hair initiation and subsequent growth are already well characterized. After the determination of the initiation site, the first morphological sign of root hair development is the appearance of a “bulge” on the outer wall of the trichoblast (Dolan et al., 1993); (Figure 1A). In *Arabidopsis thaliana*, it is followed by two phases of root hair growth: an initial period of slow growth (0.2–0.5 μm/min) and subsequent rapid growth (1–2.5 μm/min) (Dolan et al., 1994). The initial period is likely used for organizing the elements of the growth machinery to prepare the cell for the second phase: tip growth. Tip growth is a form of polar growth during which root hairs expand at the tip (Bibikova and Gilroy, 2003). In growing root hairs, the subapical region contains the endoplasmic reticulum, mitochondria, and Golgi bodies, while a large central vacuole fills the basal region (Miller et al., 2000); (Figure 1B). The extreme apex is the destination for high levels of secretory vesicles that carry the building materials for the growing plasma membrane and cell wall (Sherrier and VandenBosch, 1994; Galway et al., 1997; Miller et al., 2000). At the end of the tip-growing phase, Arabidopsis root hairs reach a final length of approximately 1 mm and are 10 μm in diameter (Grierson and Schiefelbein, 2002).

The mechanism of tip growth is complex and involves the transport of secretory vesicles toward the tip, dynamic actin and microtubule cytoskeletal networks, changes in reactive oxygen species (ROS) and Ca^2+^ gradients, as well as changes in the activity of cell wall-modifying enzymes and other processes (Mendrinna and Persson, 2015; Eljebbawi et al., 2020; Mase and Tsukagoshi, 2021). Furthermore, it has long been known that a current of protons (H^+^) enters root hairs at the tip and leaves at the base (Weisenseel et al., 1979); (Figure 1C), but its role in root hair development and growth is uncertain (Cárdenas, 2009; Schoenaers et al., 2017; Siao et al., 2020). In this review, we summarize what is known about the role of protons and pH changes in the emergence and growth of root hairs, and we highlight aspects that merit further investigation. As tip growth is not unique to root hairs, such knowledge could be useful for understanding how other tip-growing structures develop, such as pollen tubes, fungal hyphae, fucoid algal cells, moss protonemata, and nerve fibers in animals.

## pH changes during bulging: An effect or a cause?

Bulging at the trichoblast has long been known to correlate with pH changes at both the apoplast (acidification) and cytoplasm (alkalinization) (Bibikova et al., 1998; Fasano et al., 2001); (Figure 1A). A drop in pH (from ~5 to 4.5) occurs at the root hair initiation site of the cell wall. This localized cell wall acidification is not present before bulging and lasts just until the root hair reaches the tip-growing stage (Bibikova et al., 1998). When the cell wall is acidified, the cytoplasm is alkalinized, suggesting that the pH changes are due to the cellular export of protons (Bibikova et al., 1998). Inhibiting the extracellular pH drop with strong buffers prevents root hair initiation, but as the cell wall returns to acidic pH, bulging restarts. These observations have been interpreted as evidence that the pH change is the cause and not the result of cell wall bulge initiation. However, cell wall acidification alone is not sufficient to cause bulging, as lowering the pH of the entire trichoblast to 4.5 does not lead to the emergence of bulges or general cell wall swelling (Bibikova et al., 1998), which does not exclude the possibility that localized pH changes might cause bulging.

## A cytosolic pH gradient and oscillating pH

**Figure 2:**
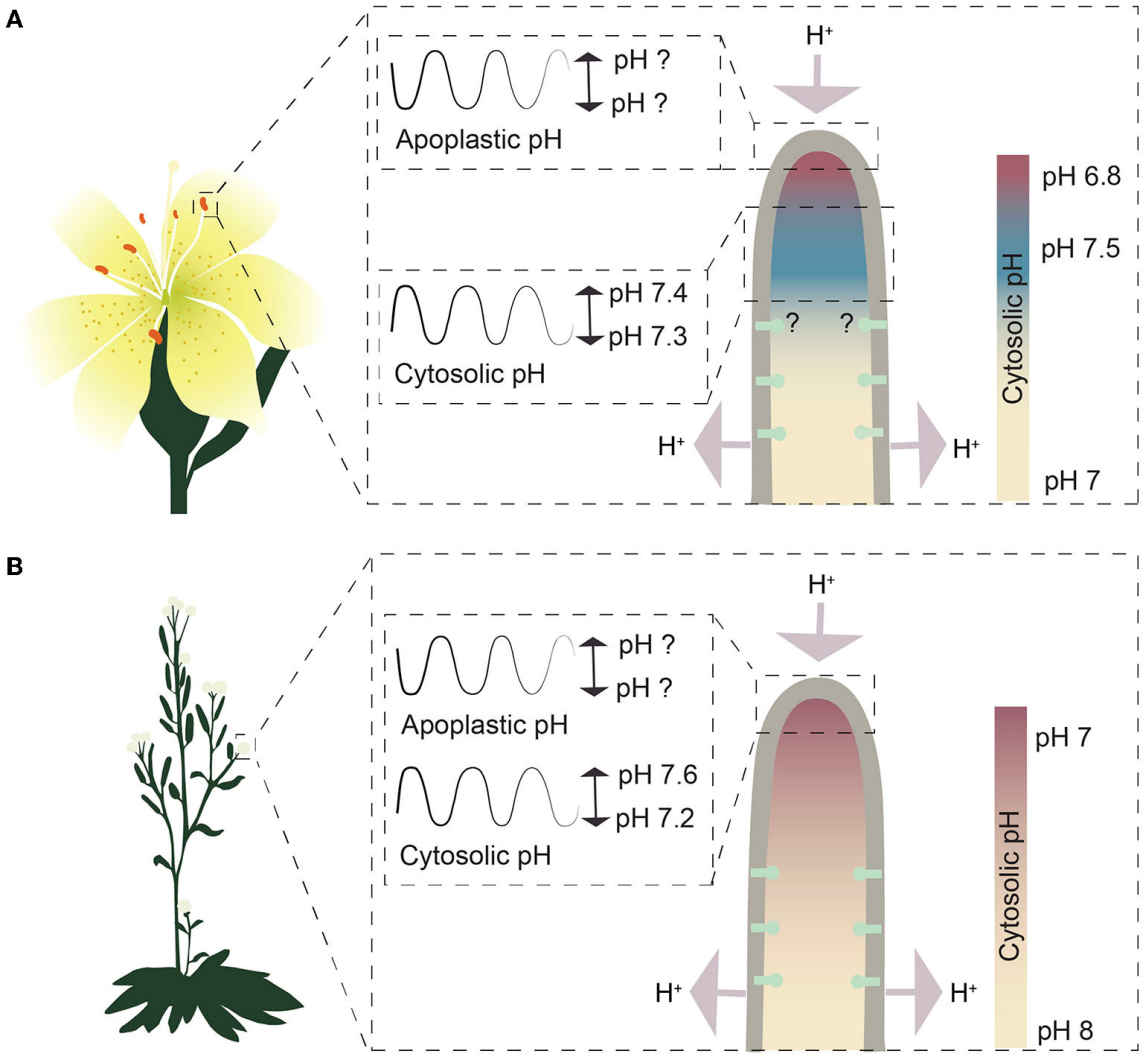
Schematic representation of growing pollen tubes from the pH point of view. **(A)** The cytosolic pH gradient and pH oscillations in growing *Lilium longiflorum* pollen tubes. Values for the pH gradient and the cytosolic pH oscillation in the alkaline band are based on measurements in Feijó et al. (1999). Question marks indicate undetermined pH values or unknown distribution of plasma membrane H^+^-ATPases. Color codes represent the cytosolic pH gradient, where red indicates slightly more acidic pH and blue more alkaline pH compared to the rest of the cytoplasm. **(B)** The cytosolic pH gradient and pH oscillations in growing Arabidopsis pollen tubes. Values for the pH gradient and the cytosolic pH oscillation are based on Hoffmann et al. (2020). Red indicates a more acidic pH, while blue indicates a more alkaline pH. Question marks indicate undetermined pH values.

Cytosolic pH can be measured with genetically encoded biosensors (Walia et al., 2018) that are less prone to artifacts associated with trapped or injected fluoroprobic dyes (Graber et al., 1986). In growing root hairs expressing a pH-sensitive ratiometric variant of *Aequorea victoria* green fluorescent protein (GFP-H148D; Elsliger et al., 1999), the cytosolic pH at the tip was measured and shown to oscillate between pH 7.4 and 7.6 (Monshausen et al., 2007); (Figure 1C). In this work, cytosolic pH at the base of root hairs was not reported. A subsequent study employed a pH-sensitive GFP from *Ptilosarcus gurneyi* (Pt-GFP; Schulte et al., 2006) and found tip cytosolic pH to be more alkaline than that at the base of root hairs (Bai et al., 2014); (Figure 1B). This observation is surprising considering that protons enter at the tip and leave the cytosol at the base (Weisenseel et al., 1979) and is different from the pH profile observed in pollen tubes, another tip-growing system. In lily (*Lilium longiflorum*) pollen tubes, cytosolic pH at the extreme apex was measured using an injected ratiometric pH sensitive dye (2′,7′-bis-(2-carboxyethyl)-5-(and-6)-carboxyfluorescein (BCECF)-dextran) and was found to be more acidic than a constitutive cytosolic alkaline band in the subapical region (Feijó et al., 1999); (Figure 2A). In this system, proton influx was revealed at the extreme apex, while efflux was reported in the region of the alkaline band (Feijó et al., 1999); (Figure 2A). Ratiometric pHluorin is another pH-sensitive GFP variant (Haseloff et al., 1997; Miesenböck et al., 1998) that has been used to image cytosolic pH in Arabidopsis pollen tubes (Hoffmann et al., 2020). This study found the cytosolic pH at the tip to be more acidic than that in the base of the pollen tube but failed to detect a subapical alkaline band (Hoffmann et al., 2020); (Figure 2B). Higher resolution cytosolic pH measurements in root hairs may resolve whether cytosolic pH at the extreme apex of root hairs is indeed acidic compared to the basal parts.

Growth analysis suggests that a consistent oscillatory root hair growth is associated with the pH oscillations at the tip with approximately two pulses per minute (Monshausen et al., 2007; Wu et al., 2007); (Figures 1C,D). These pH oscillations occur with the same periodicity as growth but out of phase. Cross-correlation analysis revealed that a decrease in extracellular pH precedes rhythmic root hair growth, whereas the maximal increase in extracellular pH lags behind growth by approximately 7 seconds (Monshausen et al., 2007); (Figure 1D). These pH oscillations are observed exclusively at the apical 5 to 10 μm of the root hair tip, not along the shanks and base, and no such changes were reported for non-growing root hairs (Monshausen et al., 2007). At the root hair apex, each extracellular alkalinization event is accompanied by transient cytosolic acidification likely due to a flux of protons from the cell wall toward the cytosol (Monshausen et al., 2007). As a result, the apoplastic pH shifts from approximately 5.4 to 5.8 and the cytosolic pH from 7.6 to 7.4; the difference in amplitude is likely due to the different buffering capacity of the apoplast and cytosol (Oja et al., 1999); (Figure 1C). There appears to be a limit to the tolerance to such pH changes, as growing root hairs tend to burst at the tip when the pH of the growth medium is artificially lowered to 4.5, while root hair growth is rapidly inhibited when the medium pH is increased to pH 8 (Monshausen et al., 2007).

These oscillatory pH changes may be required for control of actin dynamics in the growing root hair. In root hairs, long actin bundles run longitudinally along the root hair and branch out close to the tip (Cárdenas et al., 1998), and root hair growth is associated with constant polymerization of actin (Vazquez et al., 2014). Dynamic actin reorganization involves the assembly and disassembly of actin filaments that are likely regulated by pH-sensitive actin-binding proteins (Vazquez et al., 2014); (Figure 1F). In pollen tubes, alkalinization of cytosolic pH promotes actin-depolymerizing factor (ADF) protein activity so it becomes more efficient in actin depolymerization at the minus end and supports growth at the plus end (Chen et al., 2002; Lovy-Wheeler et al., 2006). It remains to be determined whether an oscillatory cytosolic pH controls actin dynamics in root hairs.

## Role of pH change in cell wall loosening

The root hair cell wall has different characteristics at the apex and in the non-expanding parts: it is thin at the growing tips and turns into a thicker, stronger, multi-layered cell wall in the shanks of root hairs (Galway, 2006). The root hair cell wall is composed mainly of (xylo)glucans, pectins, and O-glycoproteins (Galway et al., 2011; Velasquez et al., 2011; Peña et al., 2012). Lack of any of these polymers inhibits tip growth, suggesting that they operate together to control polarized growth (Bernhardt and Tierney, 2000; Favery et al., 2001; Pang et al., 2010; Ringli, 2010; Park et al., 2011; Velasquez et al., 2011; Zabotina et al., 2012; Wang et al., 2014). In response to oscillating pH changes in the tip, cell wall-loosening enzymes with different pH optima could in principle contribute to oscillating growth by alternating between loosening and rigidifying the cell wall (Schoenaers et al., 2017); (Figure 1E).

Possible cell wall-loosening enzymes in root hairs could be expansins. These proteins mediate acid-induced expansion by disrupting the bonds between cellulose and hemicellulose residues. Expansins are most active at acidic pH; for example, alpha and beta expansins (EXPs) have a pH optimum of around 4.5 (McQueen-Mason et al., 1992). EXP7 and EXP18 were detected in the cell walls of trichoblasts but not atrichoblasts, pointing to their role in root hair development (Cho and Cosgrove, 2002). Expansins were also shown to localize at the bulge site of maize (*Zea mays*) roots (Baluška et al., 2000). Their important role in root hair initiation is also supported by the finding that in barley (*Hordeum vulgare*), a root hairless mutant (*rhl1.a*) is defective in expression of the *HvEXPB1* gene, which encodes a β-expansin (Kwasniewski and Szarejko, 2006).

While the acidic pH shift activates expansins, it may actually inhibit other cell wall proteins, e.g., pectate lyases. Pectate lyases cleave glycosidic linkages with an alkaline pH optimum of ~7.5 to 8 (Ouattara et al., 2010). These enzymes are expressed in pollen tubes, and it was suggested that they are required for pectin degradation during tip growth (Wing et al., 1990). A pectate lyase, *ROOT HAIR-SPECIFIC 14* (*RHS14*), was shown to be expressed in Arabidopsis root hairs, suggesting that pectate lyases might also be involved in regulating cell wall loosening in these tubular structures (Su-Kyung et al., 2009).

Xyloglucan endotransglycosylases (XETs) are also expressed in root hairs. A highly localized up-regulation of their activity was reported exclusively at the root hair initiation site before any visible bulge formation (Vissenberg et al., 2001), which points to an important role of XETs during the initial phase. However, XET activity was uniform along the length of the growing root hair suggesting that it does not have a key role in guiding polar growth in this system. Known XETs have a pH optimum of approximately 5 to 6.5 (Han et al., 2016). However, such a pH range is higher than the reported pH 4.5 of the cell wall during bulging (Bibikova et al., 1998). Vissenberg et al. (2001) showed that this lower pH of 4.5 does not substantially affect XET activity in initiating root hairs, while no activity is detected at the initiation site if the pH is increased to 7. Therefore, it seems that XET activity is also pH dependent and that an acidophilic XET isoform accumulates at the root hair initiation site (Vissenberg et al., 2001). Accordingly, transient pH changes at the cell wall could affect cell wall dynamics.

## A putative role of plasma membrane H^+^-ATPases in root hair growth

Work on pollen tubes has shown that autoinhibited plasma membrane H^+^-ATPases (AHAs; AHA6, AHA8, and AHA9) sustain pollen tube growth, as a triple mutant deficient in these ATPases shows severe growth defects (Hoffmann et al., 2020). In pollen tubes, reverse fountain cytoplasmic streaming has been described where protons are exported at the shanks and imported at the pollen tube tip. This movement of protons likely is made possible by the presence of these plasma membrane H^+^-ATPases in the shanks and their absence from the tip (Hoffmann et al., 2020); (Figure 2B).

The first experiments pointing toward the involvement of plasma membrane H^+^-ATPases in root hair growth were performed with pharmacological inhibitors. Cyanide indirectly inhibits plasma membrane H^+^-ATPases by depleting cytosolic ATP (Lew and Spanswick, 1984), while vanadate competes with phosphate at the catalytic site of P-type ATPases, leading to direct inhibition of these pumps (Cantley et al., 1977; Sze, 1984). Both compounds inhibit root hair initiation and root hair growth (Bibikova et al., 1998). Even though these compounds are not specific for plasma membrane H^+^-ATPases, the results suggest that root hair growth is ATP dependent and is possibly carried out by P-type ATPases, a large family of primary active pumps to which plasma membrane H^+^-ATPases belong.

Of the 11 *AHAs* present in the Arabidopsis genome, *AHA1, AHA2, AHA4, AHA5, AHA7, AHA9*, and *AHA10* are expressed in root hairs (Moriau et al., 1999; Palmgren, 2001; Santi and Schmidt, 2009; Młodzińska et al., 2014). The presence of almost all *AHA* isoforms in Arabidopsis root hairs suggests a high level of redundancy. Functional redundancy of plasma membrane H^+^-ATPases has been reported previously in pollen tubes where single *aha6, aha8*, and *aha9* mutants show very mild phenotypes, whereas a triple *aha6 aha8 aha9* mutant is strongly compromised in growth (Hoffmann et al., 2020).

Strong enrichment of *AHA7* transcripts was reported in Arabidopsis root hairs that was two orders of magnitude higher in root hairs compared to other root cells (Lan et al., 2013). Furthermore, in the hairless Arabidopsis mutant *rhd2*, AHA7 was 3.6-fold less abundant than in the wild type (Jones et al., 2006). Analysis of loss of function *aha7* mutants in Arabidopsis showed that the pump is important during special conditions as the number of root hairs decreased under Fe (Santi and Schmidt, 2009) and phosphate (Yuan et al., 2017) deficiency. This would suggest that in Arabidopsis, AHA7 is involved in the root hair initiation process under limiting conditions (Santi and Schmidt, 2009). However, no significant changes in root hair length and density could be observed in *aha7* mutant lines grown under control conditions. This suggests that at least under normal conditions AHA7 serves a redundant function with other plasma membrane H^+^-ATPases (Hoffmann et al., 2019). AHA7 is unique among AHAs in that it is equipped with a pH-sensitive loop exposed to the apoplastic side of the plasma membrane, which inactivates the pump when the extracellular pH dips below pH 6.0 through protonation of an acidic group in the loop. Thus, proton export by AHA7 may be inhibited when the apoplast is acidified to a certain extent and reactivated as the apoplastic pH increases again. This could imply a mechanism for generating pH oscillations during tip growth (Hoffmann et al., 2019).

AHA2, a predominant plasma membrane H^+^-ATPase in roots (Harper et al., 1990; Fuglsang et al., 2007), is also expressed in Arabidopsis root hairs (Młodzińska et al., 2014). Loss of *AHA2* does not alter root hair density under control conditions but leads to increased root hair length, just like the lack of both *AHA2* and *AHA7* (Hoffmann et al., 2019). This phenotype is not likely to be due to overcompensation from other AHAs, as application of the P-type ATPase inhibitor vanadate at low concentrations (25 μM), which is sufficient to inhibit AHAs (O'Neill and Spanswick, 1984; Regenberg et al., 1995), also increases root hair length (Lin et al., 2015). As a decreased proton motive force would be expected to reduce nutrient uptake, stimulation of root hair growth in response to reduced plasma membrane H^+^-ATPase activity could be a developmental response to nutrient starvation. Previous observations support this hypothesis as they show that low availability of nutrients leads to longer root hairs (Bates and Lynch, 1996; Schmidt et al., 2000; López-Bucio et al., 2003; Müller and Schmidt, 2004).

Another puzzling observation is that in Arabidopsis root hairs, both AHA7 and AHA2 were equally distributed between the shanks and the tip (Fuglsang et al., 2007; Młodzińska et al., 2014; Hoffmann et al., 2019); (Figure 1C), in contrast to the distribution of plasma membrane H^+^-ATPases in pollen tubes, where they are absent from the extreme apex (Certal et al., 2008; Hoffmann et al., 2020). The presence of the proton extruding pumps at the apex seems to act against the described inside-directed proton current at the tip.

It has been suggested that expansion of root hairs and pollen tubes is driven by turgor pressure (e.g., Mendrinna and Persson, 2015), and by energizing uptake of K^+^ followed by water, plasma membrane H^+^-ATPase could play a role in this process. However, in pollen tubes, the turgor pressure was shown to remain unaltered in the *aha6 aha8 aha9* triple mutant when compared to the wild type despite a much reduced growth efficiency (Hoffmann et al., 2020). Thus, maybe other processes drive pollen tube extension, such as cytoskeleton buildup. This does not rule out the possibility that in root hairs plasma membrane H^+^-ATPases contribute to a buildup of turgor pressure. However, if this is the case, a reduced plasma membrane H^+^-ATPase activity would be expected to lead to reduced tip growth, but the opposite was observed in the *aha2 aha7* double mutant in which the root hairs are significantly longer compared to the wild type (Hoffmann et al., 2019).

## Control of H^+^ dynamics by FERONIA and RALF

**Figure 3:**
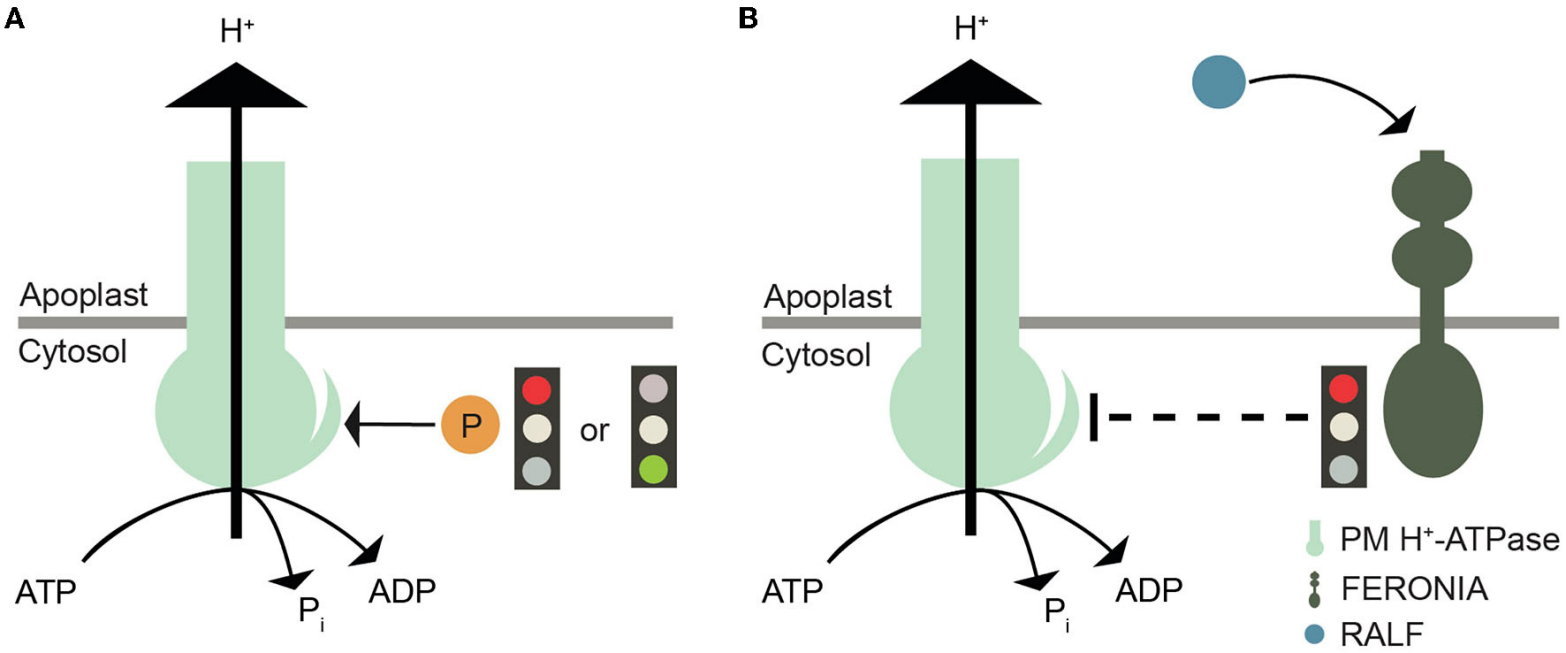
Graphical illustration of plasma membrane H^+^-ATPase regulation. **(A)** Phosphorylation of specific Thr, Ser, and Tyr residues in the C-terminal autoinhibitory domain plasma membrane H^+^-ATPases that either promote or inhibit proton efflux by this pump. **(B)** Another layer of regulation of plasma membrane H^+^-ATPases involves their inactivation by FERONIA after RALF binding.

Phosphorylation of specific Thr and Ser residues within the C-terminal regulatory domain of AHAs causes either activation or inactivation of pump activity (Falhof et al., 2016; Fuglsang and Palmgren, 2021); (Figure 3A). A number of plant receptor-like kinases (RLKs) involved in this process have been identified (Falhof et al., 2016), including FERONIA, a CrRLK1L subfamily member. FERONIA binds members of a unique peptide ligand family, RAPID ALKALINIZATION FACTOR (RALF) (Haruta et al., 2014). Following binding to the receptor at the apoplastic side of the plasma membrane, Ca^2+^ influx into the cytoplasm is initiated, with one of the downstream effects being inhibition of the plasma membrane H^+^-ATPase (Haruta et al., 2014); (Figure 3B) and, as a consequence, alkalinization of the apoplast.

In *Nicotiana attenuata*, silencing of the *Na*RALF transcript by transformation with an inverted-repeat construct of RALF results in plants with normal wild-type shoots but longer roots and trichoblasts that evolve into abnormal root hairs (Wu et al., 2007). The affected trichoblasts develop bulges that expand into spherical structures that eventually burst. The negative effect on growth is strongest at neutral pH (pH 6.8), whereas in a medium strongly buffered at a more acidic pH (pH 5.5), root hair growth partially resembles that of the wild type. Furthermore, silencing *NaRALF* results in slower extracellular pH oscillations and reduced magnitude. These pH oscillations are linked to the regulation of cell wall cross-linking, which is related to the control of tip growth (Wu et al., 2007). Taken together, these observations demonstrate that RALF has an important role in root hair development, and, since its action is associated with the alteration of proton fluxes, its effect may be linked to the inactivation of the plasma membrane H^+^-ATPase.

A role for RALF signaling in root hair development was confirmed recently following the isolation of an Arabidopsis temperature-sensitive FERONIA mutant (*fer-ts*) (Kim et al., 2021). This mutant grows like the wild type at 20°C, but at 30°C, *fer-ts* seedlings are resistant to added RALF1 peptide and do not develop root hairs. The mutant receptor carries a G41S substitution in a highly conserved glycine residue in the extracellular domain of the FERONIA receptor protein, at a position distant from the peptide binding site. Thus, the deficiency in root hair formation is probably not due to a loss of RALF1 binding as such but rather to impairment of FERONIA (Kim et al., 2021).

Root hair phenotypes of the FERONIA and RALF mutants are likely a result of crosstalk between different signaling pathways that need not involve plasma membrane H^+^-ATPase at all. For example, the RALF1-FERONIA complex phosphorylates a translation initiation factor, eIF4E1, which regulates the synthesis of root hair proteins, e.g., a Rho GTPase (ROP2) (Zhu et al., 2020) that controls root hair initiation and tip growth (Jones et al., 2002) and the transcription factor ROOT HAIR DEFECTIVE 6-LIKE 4 (RSL4) that controls the expression of hundreds of genes in root hairs (Yi et al., 2010). However, the effect of the RALF-FERONIA complex on plasma membrane H^+^-ATPase and its possible involvement in root hair growth control requires further investigation.

### The big picture: How pH changes affect ROS, Ca^2+^, and growth oscillations

Like pH, both apoplastic and cytoplasmic ROS and Ca^2+^ concentrations oscillate during root hair growth and are considered to sustain root hair tip growth (Monshausen et al., 2007); (Figure 1D). Mutants of *RESPIRATORY BURST OXIDASE HOMOLOG C* (*RBOHC*)/*ROOT HAIR DEFECTIVE 2* (*RHD2*) have short root hairs and are impaired in tip growth (Foreman et al., 2003). The encoded RBOHC/RHD2 protein is an NADPH oxidase that localizes to the root hair tip where it produces superoxide, which is converted to hydrogen peroxide (H_2_O_2_) (Foreman et al., 2003; Chapman et al., 2019). This results in a tip-focused ROS gradient in the elongating root hair (Jones et al., 2007). This tip-focused ROS gradient is hypothesized to regulate the cytoplasmic Ca^2+^ dynamics that control delivery by exocytosis of cellular material to the tip and subsequent elongation (Foreman et al., 2003). ROS act as signaling molecules in small amounts, but they may cause cellular damage and cell death at higher concentrations (Czarnocka and Karpiński, 2018). In pollen tubes, flavonols were shown to prevent ROS from accumulating at damaging levels during heat stress and thereby promote pollen tube growth even at elevated temperatures (Muhlemann et al., 2018). Consistent with flavonols acting as ROS scavengers, disrupting flavonol synthesis in tomato (*Solanum lycopersicum*) leads to higher levels of ROS in root hairs but also increased root hair formation (Maloney et al., 2014). Root hair-producing trichoblasts were shown to express ROS-producing enzymes and accumulate high levels of ROS when compared to atrichoblasts (Gayomba and Muday, 2020). Thus, the elevated levels of ROS might result in increased frequency of trichoblasts (Maloney et al., 2014).

**Figure 4:**
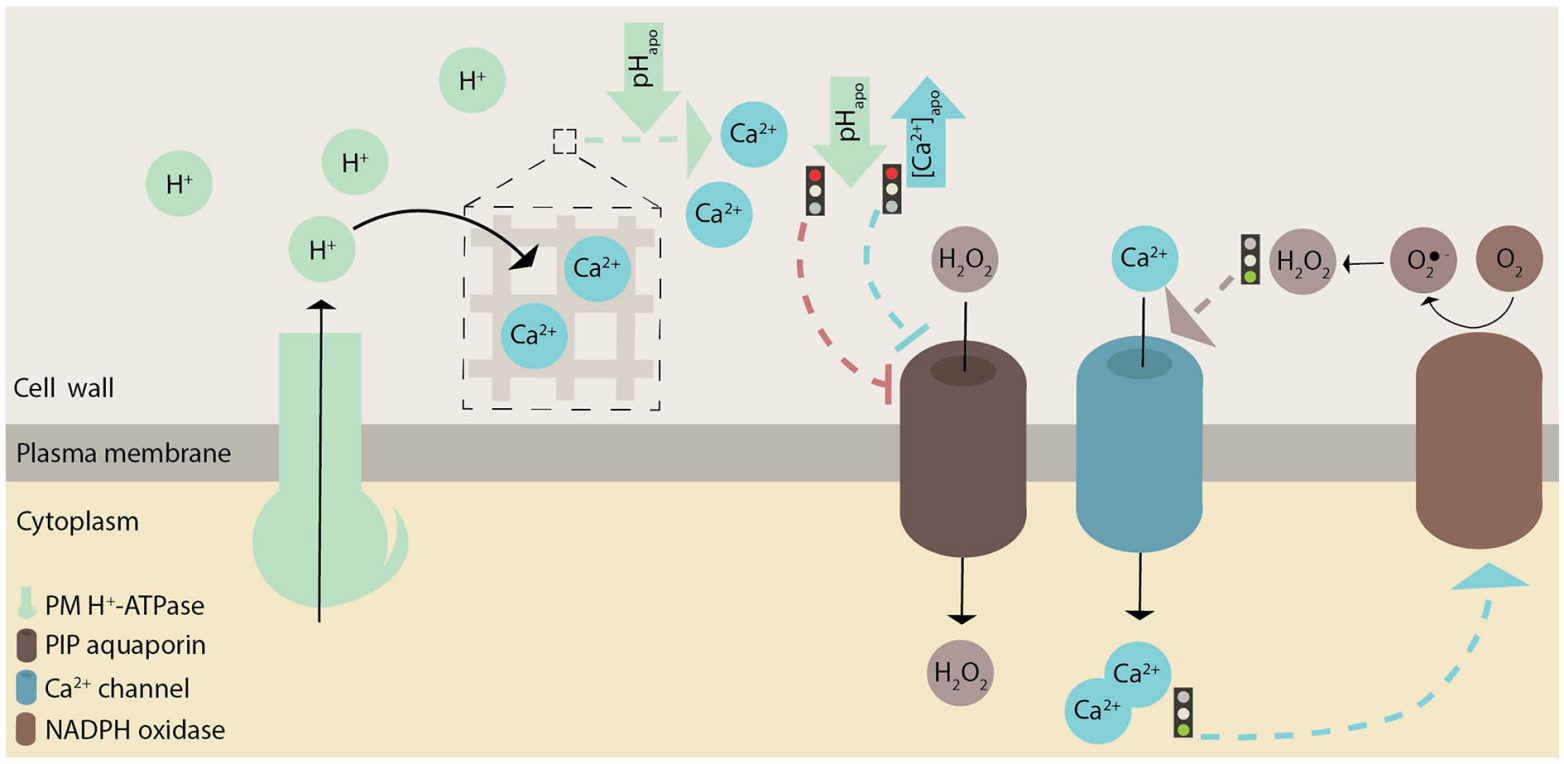
Graphical illustration of how pH, ROS, and Ca^2+^ possibly interact with each other in root hairs. These hypothesized interactions are indicated with dashed lines. In general, green represents pH, brown ROS, and blue Ca^2+^-related effects or proteins. Whether there are causative links between H^+^ and Ca^2+^ fluxes remains to be investigated.

ROS production in the apoplast seems to correlate with a cytosolic pH increase (Gonugunta et al., 2009), and an increased cytosolic Ca^2+^ concentration might promote ROS production at the root hair tip (Zhang et al., 2017); (Figure 4). ROS molecules are transported from the apoplast into the cytosol through the PLASMA MEMBRANE INTRINSIC PROTEIN (PIP) aquaporin, and this transport is inhibited by high extracellular Ca^2+^ levels and by low apoplastic pH (Byrt et al., 2017); (Figure 4). Therefore, these results suggest an association not only between Ca^2+^ and ROS, but also between pH and ROS.

Oscillating ROS levels have been proposed to contribute to a change in the active state of AHAs, and in this way ROS could control apoplastic pH oscillations (Mangano et al., 2018). However, plasma membrane H^+^-ATPases are quite insensitive to ROS due to a conserved and buried cysteine residue in close proximity to the pivotal aspartate that is phosphorylated in every catalytic cycle (Welle et al., 2021). In Arabidopsis AHA2, this Cys residue protects the pump from oxidation by ROS (Welle et al., 2021). This observation argues against a role for ROS in directly controlling plasma membrane H^+^-ATPase activities but does not exclude the possibility that plasma membrane H^+^-ATPases could be part of a mechanism that involves ROS.

However, an essential role of ROS oscillations in tip growth is challenged by the observation that a simple increase of the extracellular pH from 5 to 6 partly restored the short root hair phenotype of the *rhd2* mutant, which lacks a NADPH oxidase responsible for ROS production. How alkalinization of the growth medium results in a restored phenotype is unclear. Under these conditions, the mutant showed decreased ROS accumulation but a normal tip-focused Ca^2+^ gradient (Monshausen et al., 2007), which was supposed to be modulated by the localized accumulation of ROS. Thus, ROS may not be essential for root hair development as such and Ca^2+^ channels can be activated in another way than through ROS accumulation.

As proposed for ROS, Ca^2+^ ions function as secondary messengers. High Ca^2+^ levels trigger vesicle exocytosis and thereby the delivery of the building material of the growing root hair apex (Campanoni and Blatt, 2007). However, a direct correspondence between Ca^2+^ oscillations, vesicle secretion, and growth is not evident as the peak in Ca^2+^ oscillations follows the peak in growth rate (Monshausen et al., 2008); (Figure 1D). A concerted effect of H^+^ and Ca^2+^ is like to impact actin dynamics via Ca^2+^- and pH-sensitive actin binding proteins such as actin-depolymerizing factor (ADF), gelsolin, and villin (Ketelaar, 2013; Qian and Xiang, 2019). During Ca^2+^ oscillations, Ca^2+^ ions could be imported from the apoplast or released from subcellular components (such as vacuoles and the endoplasmic reticulum). The negatively charged pectins and arabinogalactan are capable of binding Ca^2+^ ions in the cell wall (Tian et al., 2006; Lamport and Várnai, 2013). Apoplastic pH changes mediated by plasma membrane H^+^-ATPases might regulate the release of Ca^2+^ ions from the cell wall (Virk and Cleland, 1988; Peaucelle et al., 2012); (Figure 4). The removal of Ca^2+^ from the cytoplasm requires active transport, which may be carried out by autoinhibitory P-type IIB Ca^2+^-ATPases (ACAs) or Ca^2+^/H^+^ antiporters (CAXs). Thus, H^+^ and Ca^2+^ may act together in root hair tip growth. Whether Ca^2+^ influx precedes H^+^ influx (Behera et al., 2018) or Ca^2+^ influx is lagging behind H^+^ influx (Li et al., 2021) is still a matter of debate in ion signaling and remains to be tested in root hairs.

## Conclusions and perspectives

The emergence of root hairs and their subsequent growth coincide with changes in apoplastic and cytosolic pH, which seem to involve dynamic changes in plasma membrane H^+^-ATPase activity. However, there appear to be differences with another tip-growing system, pollen tubes. In both systems, proton influx at the apex and efflux at the shank is reported. However, in root hairs, AHA2 and AHA7 proton pumps appear to be equally distributed along the root hair plasma membrane, whereas in pollen tubes a polar distribution of plasma membrane H^+^-ATPases explains mechanistically how the proton circuit can occur. We still do not understand why the cytosolic pH gradient from tip to base in root hairs and pollen tubes is apparently oriented in different directions. Moreover, it remains to be investigated what role RALF and FERONIA play in regulating plasma membrane H^+^-ATPase activity during root hair development. Furthermore, the puzzling positive effect on root hair growth exhibited by the *aha2 aha7* double knockout mutant compared to the negative effect of the *aha6 aha8 aha9* triple knockout on pollen tube growth suggests the possibility of an at least partially different mechanism for growth. The pH changes involved in root hair growth are likely a result of the crosstalk of several signaling pathways. As it stands, a broader understanding of the effect of pH on root hair growth and the role of plasma membrane H^+^-ATPases is required to develop potential general models for tip growth in diverse biological systems.

## Data availability statement

The original contributions presented in the study are included in the article/supplementary material, further inquiries can be directed to the corresponding author/s.

## Author contributions

All authors listed have made a substantial, direct, and intellectual contribution to the work and approved it for publication.

## Funding

This work was supported by grants from the Danish National Research Foundation (PUMPkin; MP) the Carlsberg Foundation (RaisingQuinoa; project number CF18-1113; MP), the Innovation Fund Denmark (LESSISMORE; MP), and the Novo Nordisk Foundation (NovoCrops; project number 2019OC53580; MP).

## Conflict of interest

The authors declare that the research was conducted in the absence of any commercial or financial relationships that could be construed as a potential conflict of interest.

## Publisher's note

All claims expressed in this article are solely those of the authors and do not necessarily represent those of their affiliated organizations, or those of the publisher, the editors and the reviewers. Any product that may be evaluated in this article, or claim that may be made by its manufacturer, is not guaranteed or endorsed by the publisher.

## References

[B1] Bai L. Ma X. Zhang G. Song S. Zhou Y. Gao L. . (2014). A receptor-like kinase mediates ammonium homeostasis and is important for the polar growth of root hairs in *Arabidopsis*. Plant Cell. 26, 1497–1511. 10.1105/tpc.114.124586 24769480 PMC4036567

[B2] Balcerowicz D. Schoenaers S. Vissenberg K. (2015). Cell fate determination and the switch from diffuse growth to planar polarity in *Arabidopsis* root epidermal cells. Front. Plant Sci. 6, 1163. 10.3389/fpls.2015.01163 PMC4688357 26779192

[B3] Baluška F. Salaj J. Mathur J. Braun M. Jasper F. Šamaj J. . (2000). Root hair formation: F-actin-dependent tip growth is initiated by local assembly of profilin-supported F-actin meshworks accumulated within expansin-enriched bulges. Dev. Biol. 227, 618–632. 10.1006/dbio.2000.9908 11071779

[B4] Bates T. R. Lynch J. P. (1996). Stimulation of root hair elongation in *Arabidopsis thaliana* by low phosphorus availability. Plant Cell Environ. 19, 529–538. 10.1111/j.1365-3040.1996.tb00386.x

[B5] Behera S. Xu Z. Luoni L. Bonza M. C. Doccula F. G. Michelis D. e. A. . (2018). Cellular Ca^2+^ signals generate defined pH signatures in plants. Plant Cell. 30, 2704–2719. 10.1105/tpc.18.00655 30377237 PMC6305977

[B6] Bernhardt C. Tierney M. L. (2000). Expression of AtPRP3, a proline-rich structural cell wall protein from *Arabidopsis*, is regulated by cell-type-specific developmental pathways involved in root hair formation. Plant Physiol. 122, 705–714. 10.1104/pp.122.3.705 10712533 PMC58905

[B7] Bibikova T. Gilroy S. (2003). Root hair development. J. Plant Growth Regul. 21, 383–415. 10.1007/s00344-003-0007-x

[B8] Bibikova T. N. Jacob T. Dahse I. Gilroy S. (1998). Localized changes in apoplastic and cytoplasmic pH are associated with root hair development in Arabidopsis thaliana. Development 125, 2925–2934. 10.1242/dev.125.15.2925 9655814

[B9] Byrt C. S. Zhao M. Kourghi M. Bose J. Henderson S. W. Qiu J. . (2017). Non-selective cation channel activity of aquaporin AtPIP2;1 regulated by Ca^2+^ and pH. Plant Cell Environ. 40, 802–815. 10.1111/pce.12832 27620834

[B10] Campanoni P. Blatt M. R. (2007). Membrane trafficking and polar growth in root hairs and pollen tubes. J. Exp. Bot. 58, 65–74. 10.1093/jxb/erl059 16873451

[B11] Cantley L. C. Josephson L. Warner R. Yanagisawa M. Lechene C. . (1977). Vanadate is a potent (Na,K)-ATPase inhibitor found in ATP derived from muscle. J. Biol. Chem. 252, 7421–7423. 10.1016/S0021-9258(17)40978-1 144127

[B12] Cárdenas L. (2009). New findings in the mechanisms regulating polar growth in root hair cells. Plant Signal. Behav. 4, 4–8. 10.4161/psb.4.1.7341 19568333 PMC2634060

[B13] Cárdenas L. Vidali L. Domínguez J. Pérez H. Sánchez F. Hepler P. K. . (1998). Rearrangement of actin microfilaments in plant root hairs responding to rhizobium etli nodulation signals. Plant Physiol. 116, 871–877. 10.1104/pp.116.3.871 9501120 PMC35089

[B14] Certal A. C. Almeida R. B. Carvalho L. M. Wong E. Moreno N. Michard E. . (2008). Exclusion of a proton ATPase from the apical membrane is associated with cell polarity and tip growth in *Nicotiana tabacum* pollen tubes. Plant Cell. 20, 614–634. 10.1105/tpc.106.047423 18364468 PMC2329945

[B15] Chapman J. M. Muhlemann J. K. Gayomba S. R. Muday G. K. (2019). RBOH-dependent ROS synthesis and ROS scavenging by plant specialized metabolites to modulate plant development and stress responses. Chem. Res. Toxicol. 32, 370–396. 10.1021/acs.chemrestox.9b00028 30781949 PMC6857786

[B16] Chen C. Y. Wong E. I. Vidali L. Estavillo A. Hepler P. K. Wu H. M. . (2002). The regulation of actin organization by actin-depolymerizing factor in elongating pollen tubes. Plant Cell. 14, 2175–2190. 10.1105/tpc.003038 12215514 PMC150764

[B17] Cho H. T. Cosgrove D. J. (2002). Regulation of root hair initiation and expansin gene expression in *Arabidopsis*. Plant Cell. 14, 3237–3253. 10.1105/tpc.006437 12468740 PMC151215

[B18] Czarnocka W. Karpiński S. (2018). Friend or foe? Reactive oxygen species production, scavenging and signaling in plant response to environmental stresses. Free Radic. Biol. Med. 122, 4–20. 10.1016/j.freeradbiomed.2018.01.011 29331649

[B19] Datta S. Kim C. M. Pernas M. Pires N. D. Proust H. Tam T. . (2011). Root hairs: development, growth and evolution at the plant-soil interface. Plant Soil. 346, 1–14. 10.1007/s11104-011-0845-422730024

[B20] Dolan L. Duckett C. M. Grierson C. Linstead P. Schneider K. Lawson E. . (1994). Clonal relationships and cell patterning in the root epidermis of *Arabidopsis*. Development 120, 2465–2474. 10.1242/dev.120.9.24659501025

[B21] Dolan L. Janmaat K. Willemsen V. Linstead P. Poethig S. Roberts K. . (1993). Cellular organisation of the *Arabidopsis thaliana* root. Development 119, 71–84. 10.1242/dev.119.1.71 8275865

[B22] Eljebbawi A. Guerrero Y. D. C. R. Dunand C. Estevez J. M. (2020). Highlighting reactive oxygen species as multitaskers in root development. iScience 24, 101978. 10.1016/j.isci.2020.101978 33490891 PMC7808913

[B23] Elsliger M. A. Wachter R. M. Hanson G. T. Kallio K. Remington S. J. (1999). Structural and spectral response of green fluorescent protein variants to changes in pH. Biochemistry 27, 5296–5301. 10.1021/bi9902182 10220315

[B24] Falhof J. Pedersen J. T. Fuglsang A. T. Palmgren M. (2016). Plasma membrane H+-ATPase regulation in the center of plant physiology. Mol. Plant 9, 323–337. 10.1016/j.molp.2015.11.002 26584714

[B25] Fasano J. M. Swanson S. J. Blancaflor E. B. Dowd P. E. Kao T. H. Gilroy S. . (2001). Changes in root cap pH are required for the gravity response of the *Arabidopsis* root. Plant Cell. 13, 907–921. 10.1105/tpc.13.4.907 11283344 PMC135544

[B26] Favery B. Ryan E. Foreman J. Linstead P. Boudonck K. Steer M. . (2001). KOJAK encodes a cellulose synthase-like protein required for root hair cell morphogenesis in Arabidopsis. Genes Dev. 15, 79–89. 10.1101/gad.188801 11156607 PMC312599

[B27] Feijó J. A. Sainhas J. Hackett G. R. Kunkel J. G. Hepler P. K. (1999). Growing pollen tubes possess a constitutive alkaline band in the clear zone and a growth-dependent acidic tip. J. Cell Biol. 144, 483–496. 10.1083/jcb.144.3.483 9971743 PMC2132912

[B28] Foreman J. Demidchik V. Bothwell J. H. Mylona P. Miedema H. . (2003). Reactive oxygen species produced by NADPH oxidase regulate plant cell growth. Nature 422, 442–446. 10.1038/nature01485 12660786

[B29] Fuglsang A. T. Guo Y. Cuin T. A. Qiu Q. Song C. Kristiansen K. A. . (2007). *Arabidopsis* protein kinase PKS5 inhibits the plasma membrane H^+^-ATPase by preventing interaction with 14-3-3 protein. Plant Cell. 19, 1617–1634. 10.1105/tpc.105.035626 17483306 PMC1913743

[B30] Fuglsang A. T. Palmgren M. (2021). Proton and calcium pumping P-type ATPases and their regulation of plant responses to the environment. Plant Physiol. 187, 1856–1875. 10.1093/plphys/kiab330 35235671 PMC8644242

[B31] Galway M. E. (2006). Root hair cell walls: filling in the framework. Botany 84, 613–621. 10.1139/b06-006

[B32] Galway M. E. Eng R. C. Schiefelbein J. W. Wasteneys G. O. (2011). Root hair-specific disruption of cellulose and xyloglucan in *AtCSLD3* mutants, and factors affecting the post-rupture resumption of mutant root hair growth. Planta 233, 985–999. 10.1007/s00425-011-1355-6 21279381

[B33] Galway M. E. Heckman J. W. Schiefelbein J. W. (1997). Growth and ultrastructure of *Arabidopsis* root hairs: the rhd3 mutation alters vacuole enlargement and tip growth. Planta 201, 209–218. 10.1007/BF01007706 9084218

[B34] Gayomba S. R. Muday G. K. (2020). Flavonols regulate root hair development by modulating accumulation of reactive oxygen species in the root epidermis. Development 147, dev185819. 10.1242/dev.185819 32179566

[B35] Gonugunta V. K. Srivastava N. Raghavendra A. S. (2009). Cytosolic alkalinization is a common and early messenger preceding the production of ROS and NO during stomatal closure by variable signals, including abscisic acid, methyl jasmonate and chitosan. Plant Signal. Behav. 4, 561–564. 10.4161/psb.4.6.8847 19816133 PMC2688314

[B36] Graber M. L. DiLillo D. C. Friedman B. L. Pastoriza-Munoz E. (1986). Characteristics of fluoroprobes for measuring intracellular pH. Anal. Biochem. 156, 202–212. 10.1016/0003-2697(86)90174-0 3740410

[B37] Grierson C. Schiefelbein J. (2002). Root hairs. Arabidopsis Book. 1, e0060. 10.1199/tab.0060 22303213 PMC3243358

[B38] Han Y. Ban Q. Hou Y. Meng K. Suo J. Rao J. . (2016). Isolation and characterization of two persimmon xyloglucan endotransglycosylase/hydrolase (XTH) genes that have divergent functions in cell wall modification and fruit postharvest softening. Front. Plant Sci. 7, 624. 10.3389/fpls.2016.00624 27242828 PMC4863071

[B39] Harper J. F. Manney L. DeWitt N. D. Yoo M. H. Sussman M. R. (1990). The *Arabidopsis thaliana* plasma membrane H^+^-ATPase multigene family. Genom sequence and expression of a third isoform. J. Biol. Chem. 265, 13601–13608. 10.1016/S0021-9258(18)77391-2 2143186

[B40] Haruta M. Sabat G. Stecker K. Minkoff B. B. Sussman M. R. (2014). A peptide hormone and its receptor protein kinase regulate plant cell expansion. Science 343, 408–411. 10.1126/science.1244454 24458638 PMC4672726

[B41] Haseloff J. Siemering K. R. Prasher D. C. Hodge S. (1997). Removal of a cryptic intron and subcellular localization of green fluorescent protein are required to mark transgenic Arabidopsis plants brightly. Proc. Natl Acad. Sci. U.S.A. 94, 2122–2127. 10.1073/pnas.94.6.2122 9122158 PMC20051

[B42] Hoffmann R. D. Olsen L. I. Ezike C. V. Pedersen J. T. Manstretta R. López-Marqués R. L. . (2019). Roles of plasma membrane proton ATPases AHA2 and AHA7 in normal growth of roots and root hairs in *Arabidopsis thaliana*. Physiol. Plant. 166, 848–861. 10.1111/ppl.12842 30238999 PMC7379730

[B43] Hoffmann R. D. Portes M. T. Olsen L. I. Damineli D. S. C. Hayashi M. Nunes C. O. . (2020). Plasma membrane H^+^-ATPases sustain pollen tube growth and fertilization. Nat. Commun. 11, 2395. 10.1038/s41467-020-16253-1 PMC7224221 32409656

[B44] Jones M. A. Raymond M. J. Smirnoff N. (2006). Analysis of the root-hair morphogenesis transcriptome reveals the molecular identity of six genes with roles in root-hair development in *Arabidopsis*. Plant J. 45, 83–100. 10.1111/j.1365-313X.2005.02609.x 16367956

[B45] Jones M. A. Raymond M. J. Yang Z. Smirnoff N. (2007). NADPH oxidase-dependent reactive oxygen species formation required for root hair growth depends on ROP GTPase. J. Exp. Bot. 58, 1261–1270. 10.1093/jxb/erl279 17301029

[B46] Jones M. A. Shen J. J. Fu Y. Li H. Yang Z. Grierson C. S. . (2002). The arabidopsis Rop2 GTPase is a positive regulator of both root hair initiation and tip growth. Plant Cell 14, 763–776. 10.1105/tpc.010359 11971133 PMC150680

[B47] Ketelaar T. (2013). The actin cytoskeleton in root hairs: all is fine at the tip. Curr. Opin. Plant Biol. 16, 749–56. 10.1016/j.pbi.2013.10.003 24446547

[B48] Kim D. Yang J. Gu F. Park S. Combs J. Adams A. . (2021). A temperature-sensitive FERONIA mutant allele that alters root hair growth. Plant Physiol. 185, 405–423. 10.1093/plphys/kiaa051 33721904 PMC8133571

[B49] Kwasniewski M. Szarejko I. (2006). Molecular cloning and characterization of β-expansin gene related root hair formation in barley. Plant Physiol. 141, 1149–1158. 10.1104/pp.106.078626 16679418 PMC1489888

[B50] Lamport D. T. A. Várnai P. (2013). Periplasmic arabinogalactan glycoproteins act as a calcium capacitor that regulates plant growth and development. New Phytol. 197, 58–64. 10.1111/nph.12005 23106282

[B51] Lan P. Li W. Lin W. D. Santi S. (2013). Mapping gene activity of *Arabidopsis* root hairs. Genome Biol. 14, R67. 10.1186/gb-2013-14-6-r67 23800126 PMC3707065

[B52] Lew R. R. Spanswick R. M. (1984). Characterization of electrogenicity of soybean (*Glycine max L*.) roots. Plant Physiol. 75, 1–6. 10.1104/pp.75.1.1 16663550 PMC1066824

[B53] Li K. Prada J. Damineli D. S. C. Liese A. Romeis T. Dandekar T. . (2021). An optimized genetically encoded dual reporter for simultaneous ratio imaging of Ca^2+^ and H^+^ reveals new insights into ion signaling in plants. New Phytol. 230, 2292–2310. 10.1111/nph.17202 33455006 PMC8383442

[B54] Lin C. Y. Huang L. Y. Chi W. C. Huang T. L. Kakimoto T. Tsai C. R. . (2015). Pathways involved in vanadate-induced root hair formation in Arabidopsis. Physiol. Plant. 153, 137–148. 10.1111/ppl.12229 24833217

[B55] López-Bucio J. Cruz-Ramirez A. Herrera-Estrella L. (2003). The role of nutrient availability in regulating root architecture. Curr. Opin. Plant Biol. 6, 280–287. 10.1016/S1369-5266(03)00035-9 12753979

[B56] Lovy-Wheeler A. Kunkel J. G. Allwood E. G. Hussey P. J. Hepler P. K. (2006). Oscillatory increases in alkalinity anticipate growth and may regulate actin dynamics in pollen tubes of lily. Plant Cell 18, 2182–2193. 10.1105/tpc.106.044867 16920777 PMC1560910

[B57] Maloney G. S. DiNapoli K. T. Muday G. K. (2014). The anthocyanin reduced tomato mutant demonstrates the role of flavonols in tomato lateral root and root hair Development 166, 614–631. 10.1104/pp.114.240507 25006027 PMC4213093

[B58] Mangano S. Martínez-Pacheco J. Buslje C. M. (2018). How does pH fit in with oscillating polar growth? Trends Plant Sci. 23, 479–489. 10.1016/j.tplants.2018.02.008 29605100

[B59] Mase K. Tsukagoshi H. (2021). Reactive oxygen species link gene regulatory networks during *Arabidopsis* root development. Front. Plant Sci. 12, 660274. 10.3389/fpls.2021.660274 PMC8110921 33986765

[B60] Masucci J. D. Rerie W. G. Foreman D. R. Zhang M. Galway M. E. Marks M. D. . (1996). The homeobox gene GLABRA2 is required for position-dependent cell differentiation in the root epidermis of *Arabidopsis thaliana*. Development 122, 1253–1260. 10.1242/dev.122.4.1253 8620852

[B61] McQueen-Mason S. Durachko D. M. Cosgrove D. J. (1992). Two endogenous proteins that induce cell wall extension in plants. Plant Cell 4, 1425–1433. 10.1105/tpc.4.11.1425 11538167 PMC160229

[B62] Mendrinna A. Persson S. (2015). Root hair growth: it's a one way street. F1000Prime Rep. 7, 23. 10.12703/P7-23 PMC4335795 25750741

[B63] Miesenböck G. De Angelis D. Rothman D. A. J. E. (1998). Visualizing secretion and synaptic transmission with pH-sensitive green fluorescent proteins. Nature 394, 192–195. 10.1038/28190 9671304

[B64] Miller D. D. Leferink-ten Klooster H. B. Emons A. M. (2000). Lipochito-oligosaccharide nodulation factors stimulate cytoplasmic polarity with longitudinal endoplasmic reticulum and vesicles at the tip in vetch root hairs. Mol. Plant Microbe Interact. 13, 1385–1390. 10.1094/MPMI.2000.13.12.1385 11106032

[B65] Młodzińska E. Kłobus G. Christensen M. D. Fuglsang A. T. (2014). The plasma membrane H^+^-ATPase AHA2 contributes to the root architecture in response to different nitrogen supply. Physiol. Plant. 154, 270–282. 10.1111/ppl.12305 25382626

[B66] Monshausen G. B. Bibikova T. N. Messerli M. A. Shi C. Gilroy S. (2007). Oscillations in extracellular pH and reactive oxygen species modulate tip growth of *Arabidopsis* root hairs. Proc. Natl. Acad. Sci. U.S.A. 104, 20996–21001. 10.1073/pnas.0708586104 18079291 PMC2409255

[B67] Monshausen G. B. Messerli M. A. Gilroy S. (2008). Imaging of the Yellow Cameleon 3.6 indicator reveals that elevations in cytosolic Ca^2+^ follow oscillating increases in growth in root hairs of *Arabidopsis*. Plant Physiol. 147, 1690–1698. 10.1104/pp.108.123638 18583529 PMC2492656

[B68] Moriau L. Michelet B. Bogaerts P. Lambert L. Michel A. Oufattole M. . (1999). Expression analysis of two gene sub-families encoding the plasma membrane H^+^-ATPase in *Nicotiana plumbaginifolia* reveals the major transport functions of this enzyme. Plant J. 19, 31–41. 10.1046/j.1365-313X.1999.00495.x 10417724

[B69] Muhlemann J. K. Younts T. L. B. Muday G. K. (2018). Flavonols control pollen tube growth and integrity by regulating ROS homeostasis during high-temperature stress. Proc. Natl. Acad. Sci. U.S.A. 115, E11188–E11197. 10.1073/pnas.1811492115 30413622 PMC6255205

[B70] Müller M. Schmidt W. (2004). Environmentally induced plasticity of root hair development in *Arabidopsis*. Plant Physiol. 134, 409–419. 10.1104/pp.103.029066 14730071 PMC371035

[B71] Oja V. Savchenko G. Jakob B. Heber U. (1999). pH and buffer capacities of apoplastic and cytoplasmic cell compartments in leaves. Planta 209, 239–249. 10.1007/s004250050628 10436227

[B72] O'Neill S. D. Spanswick R. M. (1984). Effects of vanadate on the plasma membrane ATPase of red beet and corn. Plant Physiol. 75, 586–591. 10.1104/pp.75.3.586 16663670 PMC1066959

[B73] Ouattara H. G. Reverchon S. Niamke S. L. Nasser W. (2010). Biochemical properties of pectate lyases produced by three different Bacillus strains isolated from fermenting Cocoa beans and characterization of their cloned genes. Appl. Environ. Microbiol. 76, 5214–5220. 10.1128/AEM.00705-10 20543060 PMC2916476

[B74] Palmgren M. G. (2001). Plant plasma membrane H^+^-ATPases: Powerhouses for nutrient uptake. Annu. Rev. Plant Physiol. Plant Mol. Biol. 52, 817–845. 10.1146/annurev.arplant.52.1.817 11337417

[B75] Pang C. Y. Wang H. Pang Y. Xu C. Jiao Y. Qin Y. M. . (2010). Comparative proteomics indicates that biosynthesis of pectic precursors is important for cotton fiber and *Arabidopsis* root hair elongation. Mol. Cell. Proteomics 9, 2019–2033. 10.1074/mcp.M110.000349 20525998 PMC2938120

[B76] Park S. Szumlanski A. L. Gu F. Guo F. Nielsen E. (2011). A role for CSLD3 during cell-wall synthesis in apical plasma membranes of tip-growing root-hair cells. Nat. Cell Biol. 13, 973–980. 10.1038/ncb2294 21765420

[B77] Peaucelle A. Braybrook S. Höfte H. (2012). Cell wall mechanics and growth control in plants: the role of pectins revisited. Front. Plant Sci. 3, 121. 10.3389/fpls.2012.00121 PMC3368173 22685449

[B78] Peña M. J. Kong Y. York W. S. O'Neill M. A. (2012). A galacturonic acid–containing xyloglucan is involved in *Arabidopsis* root hair tip growth. Plant Cell 24, 4511–4524. 10.1105/tpc.112.103390 23175743 PMC3531849

[B79] Qian D. Xiang Y. (2019). Actin cytoskeleton as actor in upstream and downstream of calcium signaling in plant cells. Int. J. Mol. Sci. 20, 1403. 10.3390/ijms20061403 30897737 PMC6471457

[B80] Regenberg B. Villalba J. M. Lanfermeijer F. C. Palmgren M. G. (1995). C-terminal deletion analysis of plant plasma membrane H^+^-ATPase: Yeast as a model system for solute transport across the plant plasma membrane. Plant Cell 7, 1655–1666. 10.1105/tpc.7.10.1655 7580256 PMC161027

[B81] Ringli C. (2010). Monitoring the outside: cell wall-sensing mechanisms. Plant Physiol. 153, 1445–1452. 10.1104/pp.110.154518 20508141 PMC2923904

[B82] Santi S. Schmidt W. (2009). Dissecting iron deficiency-induced proton extrusion in *Arabidopsis* roots. New Phytol. 183, 1072–1084. 10.1111/j.1469-8137.2009.02908.x 19549134

[B83] Schmidt W. Tittel J. Schikora A. (2000). Role of hormones in the induction of iron deficiency responses in *Arabidopsis* roots. Plant Physiol. 122, 1109–1118. 10.1104/pp.122.4.1109 10759506 PMC58945

[B84] Schoenaers S. Balcerowicz D. Vissenberg K. (2017). “Molecular mechanisms regulating root hair tip growth: a comparison with pollen tubes,” in Pollen Tip Growth, eds G. Obermeyer and J. Feijó (Cham: Springer). 10.1007/978-3-319-56645-0_9

[B85] Schulte A. Lorenzen I. Böttcher M. Plieth C. (2006). A novel fluorescent pH probe for expression in plants. Plant Methods 2, 7. 10.1186/1746-4811-2-7 16600023 PMC1475855

[B86] Sherrier D. J. VandenBosch K. A. (1994). Secretion of cell wall polysaccharides in *Vicia* root hairs. Plant J. 5, 185–195. 10.1046/j.1365-313X.1994.05020185.x

[B87] Siao W. Coskun D. Baluška F. Kronzucker H. J. Xu W. (2020). Root-apex proton fluxes at the center of soil-stress acclimation. Trends Plant Sci. 25, 794–804. 10.1016/j.tplants.2020.03.002 32673580

[B88] Su-Kyung W. Yong-Ju L. Ha-Yeon L. Yoon-Kyung H. Misuk C. Hyung-Taeg C. . (2009). Cis-element- and transcriptome-based screening of root hair-specific genes and their functional characterization in *Arabidopsis*. Plant Physiol. 150, 1459–1473. 10.1104/pp.109.140905 19448035 PMC2705046

[B89] Sze H. (1984). H^+^-translocating ATPases of the plasma membrane and tonoplast of plant cells. Physiol. Plant. 61, 683–691. 10.1111/j.1399-3054.1984.tb05191.x20632185

[B90] Tian G. W. Chen M. H. Zaltsman A. Citovsky V. (2006). Pollen-specific pectin methylesterase involved in pollen tube growth. Dev. Biol. 294, 83–91. 10.1016/j.ydbio.2006.02.026 16564517

[B91] Vazquez L. A. B. Sanchez R. Hernandez-Barrera A. Zepeda-Jazo I. Sánchez F. Quinto C. . (2014). Actin polymerization drives polar growth in Arabidopsis root hair cells. Plant Signal. Behav. 9, e29401. 10.4161/psb.29401 25763621 PMC4203500

[B92] Velasquez S. M. Ricardi M. M. Dorosz J. G. Fernandez P. V. Nadram A. D Pol-Fachin L. . (2011). O-glycosylated cell wall proteins are essential in root hair growth. Science 332, 1401–1403. 10.1126/science.1206657 21680836

[B93] Virk S. S. Cleland R. (1988). Calcium and the mechanical properties of soybean hypocotyl cell walls: possible role of calcium and protons in cell-wall loosening. Planta 176, 60–67. 10.1007/BF00392480 24220735

[B94] Vissenberg K. Fry S. C. Verbelen J. P. (2001). Root hair initiation is coupled to a highly localized increase of xyloglucan endotransglycosylase action in *Arabidopsis* roots. Plant Physiol. 127, 1125–1135. 10.1104/pp.010295 11706192 PMC129281

[B95] Walia A. Waadt R. Jones A. M. (2018). Genetically encoded biosensors in plants: pathways to discovery. Annu. Rev. Plant Biol. 69, 497–524. 10.1146/annurev-arplant-042817-040104 29719164

[B96] Wang S. Li E. Porth I. Chen J. G. Mansfield S. D. Douglas C. J. . (2014). Regulation of secondary cell wall biosynthesis by poplar R2R3 MYB transcription factor PtrMYB152 in *Arabidopsis. Sci. Rep*. 4, 5054. 10.1038/srep05054 PMC4031478 24852237

[B97] Weisenseel M. H. Dorn A. Jaffe L. F. (1979). Natural H^+^ currents traverse growing roots and root hairs of barley (*Hordeum vulgare* L.). *Plant Physiol*. 64, 512–518. 10.1104/pp.64.3.512 16661000 PMC543125

[B98] Welle M. Pedersen J. T. Ravnsborg T. Hayashi M. Maa S. Becher D. . (2021). A conserved, buried cysteine near the P-site is accessible to cysteine modifications and increases ROS stability in the P-Type plasma membrane H^+^-ATPase. Biochemistry 478, 619–632. 10.1042/BCJ20200559 33427868

[B99] Wing R. A. Yamaguchi J. Larabell S. K. Ursin V. M. McCormick S. (1990). Molecular and genetic characterization of two pollen-expressed genes that have sequence similarity to pectate lyases of the plant pathogen Erwinia. Plant Mol. Biol. 14, 17–28. 10.1007/BF00015651 1983191

[B100] Wu J. Kurten E. L. Monshausen G. Hummel G. M. Gilroy S. Baldwin I. T. . (2007). NaRALF, a peptide signal essential for the regulation of root hair tip apoplastic pH in *Nicotiana attenuate*, is required for root hair development and plant growth in native soils. Plant J. 52, 877–890. 10.1111/j.1365-313X.2007.03289.x 17916115

[B101] Yi K. Menand B. Bell E. Dolan L. (2010). A basic helix-loop-helix transcription factor controls cell growth and size in root hairs. Nat. Genet. 42, 264–267. 10.1038/ng.529 20139979

[B102] Yuan W. Zhang D. Song T. Xu F. Lin S. Xu W. . (2017). *Arabidopsis* plasma membrane H^+^-ATPase genes AHA2 and AHA7 have distinct and overlapping roles in the modulation of root tip H^+^ efflux in response to low-phosphorus stress. J. Exp. Bot. 68, 1731–1741. 10.1093/jxb/erx040 28369625 PMC5441905

[B103] Zabotina O. A. Avci U. Cavalier D. Pattathil S. Chou Y. H. Eberhard S. . (2012). Mutations in multiple XXT genes of *Arabidopsis* reveal the complexity of xyloglucan biosynthesis. Plant Physiol. 159, 1367–1384. 10.1104/pp.112.198119 22696020 PMC3425184

[B104] Zhang D. J. Yang Y. Liu C. Y. Wu Q. S. (2017). “Reactive oxygen species signaling and root hair development: boon or bane-revisiting the role of ROS,” in Reactive Oxygen Species in Plants, eds V. P. Singh, S. Singh, D. K. Tripathi, S. M. Prasad, D. K. Chauhan (Hoboken: John Wiley and Sons). 10.1002/9781119324928.ch16

[B105] Zhu S. Estévez J. M. Liao H. Zhu Y. Yang T. Li C. . (2020). The RALF1-FERONIA complex phosphorylates eIF4E1 to promote protein synthesis and polar root hair growth. Mol. Plant. 5, 698–716. 10.1016/j.molp.2019.12.014 31904511

